# Foot Bionics Research Based on Reindeer Hoof Attachment Mechanism and Macro/Microstructures

**DOI:** 10.3390/biomimetics8080600

**Published:** 2023-12-12

**Authors:** Guoyu Li, Rui Zhang, Yexuan Luo, Yue Liu, Qiang Cao, Jiafeng Song

**Affiliations:** 1 School of Mechanical Engineering, Shanghai Dianji University, Shanghai 201306, China; ligy@sdju.edu.cn (G.L.); 15256675901@163.com (Y.L.); liuyue@sdju.edu.cn (Y.L.); caoq@sdju.edu.cn (Q.C.); 2 Key Laboratory of Bionic Engineering, Ministry of Education, Jilin University, Changchun 130025, China; 3 State Key Laboratory Automotive Safety and Energy, Tsinghua University, Beijing 100084, China; iansongjiafeng@163.com

**Keywords:** reindeer hoof, bionic feet, macro/microstructures, finite element simulation, attachment mechanism

## Abstract

The attachment performances of mechanical feet are significant in improving the trafficability and mobility of robots on the extreme ground. In the future, frozen-ground robots can be used to replace human soldiers in scouting and deep space exploration. In this study, the influence factors on the attachment function of the bionic feet were analyzed. Soft frozen soil and tight frozen soil close to natural frozen soil were prepared, and the friction between ungula and frozen soil ground was simulated together with the plantar pressures of reindeer under trotting. The major attachment parts were the ungula cusp, outer edges, and ungula capsules, and the stress on the ungula was mainly 4.56–24.72 MPa. According to the microstructures of plantar fur and ungula, the corresponding ratio of the rib width and length was 0.65:1, and the corresponding ratio of the rib width and distance was 3:1. In addition, the scales of the plantar fur were very tightly arranged and had large ripples. Based on typical curves, an ungula capsule-curved surface, and a nonsmooth plantar fur surface, four types of bionic feet and the corresponding ordinary multidamboard foot were designed. On the frozen soil, the bionic foot with ribs and an ungula capsule showed the best attachment performance. Compared with the multidamboard foot, the dynamic coefficient of friction of the bionic foot with ribs and ungula capsules increased by 11.43–31.75%. The attachment mechanism of the bionic feet is as follows: under the action of pressure, the fine patterns of the bionic convex-crown generate friction with the nonsmooth structure of the frozen soil surface, which improves the attachment performance.

## 1. Introduction

With the development of artificial intelligence, robots have gradually entered daily life to replace humans in manufacturing. For example, Atlas and Digit robots are capable of logistics and package delivery [1,2]. Unmanned military equipment has become an inevitable trend, and various military robots are becoming more and more widely used. For instance, Packbot robots can complete reconnaissance, exploration, and disposal of explosive devices [3]. The end of a robotic foot is the only part contacting the ground, and it is the main part that affects stability and traction with the ground.

Bionic-legged robots have high movement flexibility and environmental adaptability on unconventional ground due to their discrete point contacts and flexible limb structures. They have been used in exploration and transportation in high-risk environments, such as frozen ground [4]. BigDog, the military quadruped robot developed by Boston Dynamics, is equipped with advanced controllers (e.g., terrain sensors, dynamic control) to coordinate leg movements and ensure movement balance and stability. Its controllers adapt to terrain changes through terrain measurement and attitude control [5]. Researchers from MIT analyzed the kinetics, energy consumption, and speed of quadrupeds during movement and designed a bionic, lightweight mechanical foot simulating a cheetah hindlimb based on the principle of tendon-bone copositioning. This mechanical foot reduces the weight while meeting the premise of stiffness and strength, avoids large bending moments, and improves energy efficiency, which contributes to high-speed movement [6]. The second-generation Atlas has made breakthroughs in portability and balance. The internal sensors and lidar positioning allow this robot to avoid obstacles, maintain balance, and complete movements outdoors [7]. However, the robot will still slip upon walking on the snow and can only collect posture data through sensors and adjust its motion to keep its body balanced. Due to the control algorithm, image measurement and gait adjustment endow the foot robot with anti-interference ability and the ability to walk on frozen ground to a certain extent. However, the robot is limited by low walking stability and a relatively simple foot structure, and there is little research on the bionic design of bionic feet on frozen ground.

Frozen soils, as a type of extreme ground, are widely distributed around the world. The total area of frozen soils in the world is approximately 35 million km^2^, accounting for 25% of the entire earth’s area. Frozen soils are more widely found in the northern hemisphere. In recent decades, as scientists have explored the moon, they have discovered that water ice exists on the moon, but the specific location and content of water ice are still at the research stage [8]. NASA and ESA carried out lunar water ice detection in the permanent shadow areas of the North and South Poles using lunar probes, such as Clementine, Lunar Prospector, and SMART-1, and initially concluded that water ice may exist [9,10,11]. Additionally, LCROSS and LRO cooperated in impacting the moon. Through thermal imaging, near-infrared spectroscopy, ultraviolet spectroscopy, and other technologies, they detected water vapor in the impact sputters and successfully proved the existence of water ice on the moon [12]. In 2010, the Mini-Star radar mounted on Chandrayaan-1 detected more than 40 craters containing water ice at the lunar north pole. It was estimated that the content of water ice was approximately 0.6 billion tons [13]. Lunar water ice is not only a hot spot for international exploration, but also a future strategic resource. At present, all the major global space powers have formulated plans to detect and sample lunar water ice. ESA has proposed a plan to build a lunar village. China plans to conduct multiple detections and samples of lunar water ice by 2030 [14]. Lunar water ice mainly exists in permanent shadow areas (craters), which are difficult for wheeled robots to pass through. In comparison, footed robots have good application prospects owing to their excellent active adaptability and the attachment of the foot [15].

Under the law of natural selection, the foot structures, movements, and functions of animals have evolved to adapt to the environment. The engineering application of special structures of animal feet based on bionics has long been a source for solving problems and finding inspiration [16]. Mechanical feet designed with hoofed animals as bionic prototypes show excellent adaptability and movement characteristics on various grounds. Abad designed a goat-hoof-like mechanical foot with flexible joints, strong attachment, and cushioning vibration reduction. The hoof anterior and ungula capsule patterns of the mechanical foot improve attachment performance by embedding into the ground and enlarging the contact area, respectively. The pattern structure is stuck in the rock or soil and can provide an additional braking effect [17]. Horse feet have mobility, load-bearing capacity, and endurance characteristics during movement. Garcia simulated the horse muscle system by connecting elastic elements and excitation units in series and designed a bionic mechanical foot based on effective leg length, kinematics, and foot mass distribution. The key factors that influenced the agility of the mechanical foot were mentioned in [18].

**Figure 1:**
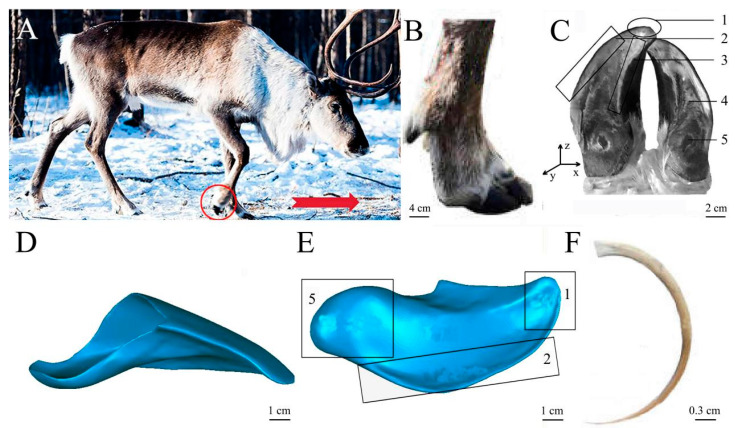
Reindeer sample and ungula model. (**A**) Reindeer; (**B**) lateral view of ungula; (**C**) plantar view of ungula; (**D**) lateral view of 3D ungula model; (**E**) plantar view of 3D ungula model; (**F**) fur of planta pedis. 1—ungula cusp, 2—ungula outer edge, 3—ungula inner edge, 4—ungula sole, 5—ungula capsule.

Reindeer (*Rangifer tartandus*), as a typical polar migratory animal, belongs to the Cervidae, Artiodactyla family, and has a hoof structure suitable for migration in complex environments [19]. In particular, reindeer hooves have good traction on frozen ground [20]. The ungula structure and plantar fur can enlarge the contact area with the ground and play an antislip role when walking on frozen ground (Figure 1). Reindeer migrate seasonally over long distances on land, and some populations migrate farther than other terrestrial mammals [21]. The gait and limb sequences of reindeer are classified into walking and trotting. According to our previous research, the kinematics and vertical ground reaction force (GRF) of reindeer forelimb joints under walking and trotting were measured using a motion tracking system and Footscan pressure plates. The study found that, compared to typical ungulates, reindeer toe joints exhibit greater stability, and the stability and energy storage in the forelimb joints contribute to the locomotor performance of reindeer. In trotting gait, one forelimb and its diagonal hindlimb move together, and only two limbs are in the stance phase (sometimes all four limbs may be in the swing phase simultaneously, e.g., the leaving sequence of left hind and right fore-right hind and left fore) [22]. Additionally, the plantar pressure distribution during trotting for the reindeer forefeet, which we have not yet published.

We selected the front hooves of reindeer and, in combination with the attachment characteristics of the hoof stance unit, conducted research on the attachment, including the ungula structure and plantar fur. Based on the reindeer hooves and together with the macro/microstructures and attachment mechanism of the stance unit, we designed the bionic mechanical feet, which provide researchers with new ideas for designing the attachment function of mechanical feet. Through foot attachment tests, we discussed the attachment mechanism between the bionic foot and the frozen soil.

## 2. Simulation Analysis of Attachment between Reindeer Ungula and Frozen Soil

### 2.1. Sample Selection

Four reindeer front hooves were selected from the Ewenki ethnic group in Genhe City, China (Figure 1A). Each reindeer was an adult male reindeer that died naturally. The weight, shoulder width, and body length of the reindeer were 118.75 ± 14.93 kg, 1.22 ± 0.51 m, and 1.89 ± 0.83 m, respectively (mean ± standard deviation). The hooves were free of disease or had not undergone any surgical treatment or other invasive procedures (Figure 1B,C). Before the experiments, the reindeer hooves were washed with distilled water to remove surface dirt from the samples. The samples were naturally dried and then scanned with CT (220 KV, 220 MA, 1.25 mm layer thickness).

### 2.2. Three-Dimensional Modeling and Reconstruction of the Ungula

Ungula is irregular, and its force acting on the ground can be hardly measured through field tests. Inverse reconstruction technology and mechanical data can be used to study the influence of reindeer ungula morphology on the attachment characteristics of frozen ground during trotting through finite element analysis. The CT-scanned hoof model was mesh-healed, relaxed, and smoothed in Geomagic Studio to generate a 3D model (Figure 1D,E). In addition, the fur of planta pedis was also in direct contact with frozen soil (Figure 1F).

### 2.3. Plantar Pressure of the Forefeet during Trotting

**Figure 2:**
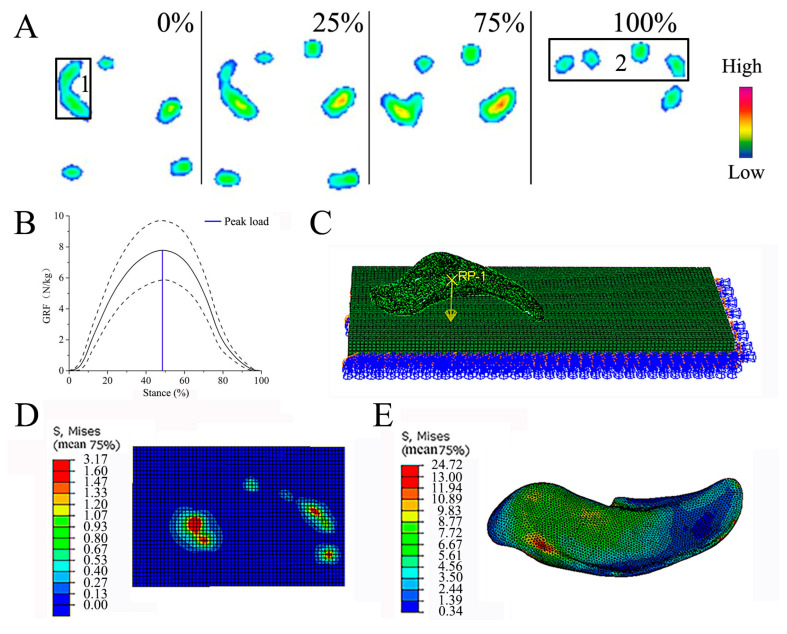
Friction simulation conditions and results. (**A**) The plantar pressure distribution during trotting for the reindeer forefeet in the 0%, 25%, 75%, and 100% stance phase; (**B**) plantar pressure during trotting for the reindeer forefeet [22]; (**C**) simulation conditions of ungula and frozen soil; (**D**) stress distribution on the surface of frozen soil; (**E**) stress distribution on the surface of ungula. 1—ungula edge and ungula capsule, 2—ungula cusp.

According to our previous research [22], during trotting on a hard surface, the forefeet made simultaneous contact with the ground through the ungula. The area of the ungula edge and ungula capsule exhibited high pressure, indicating that the areas bore the most weight. When the reindeer moved, the ungula was in contact with the ground at the early stance stage. At this time, the parts of the ungula that played a role in the load-bearing stage were mainly the ungula cusp, the ungula outer edges, and the ungula capsule. In the latter stance stage, only the ungula cusp was in contact with the ground. At this time, the hooves were pushing on the ground, and the parts that performed the attachment function were mainly the ungula cusp (Figure 2A). Therefore, the ungula cusp, ungula outer edges, and ungula capsule contribute to traction on frozen ground. Measurement of reindeer plantar pressure during trotting showed the peak load of plantar pressure was 302.93 N, which was used as the load for simulation (Figure 2B).

### 2.4. Friction Simulation Parameters and Conditions

The ungula was divided into tetrahedral meshes in the optistruct module of hypermesh. After unit size adjustment and splitting, deformation-free C3D4 unit meshes were obtained. The length, width, and height of the frozen soil were 200, 80, and 10 mm, respectively, and the frozen soil was divided into hexahedral meshes. In Abaqus, the dynamic step was used to simulate the friction process between the ungula and frozen soil. Based on the mechanical properties of frozen soil and the material measurement of ungula, the material values were assigned to the frozen soil and ungula models. The densities, elastic moduli, and Poisson ratios of the frozen soil (ungula) were 1.80 × 10^−9^ (1.15 × 10^−9^) t/mm^3^, 34.12 (505.78) MPa, and 0.29 (0.30), respectively.

The traction speed was 500 mm/min, and the friction coefficient was 0.7 (Figure 2C). Shahkhosravi and Magdalena, in their simulation study on horse’s hoof friction, utilized coefficients of friction values comparable to those in our study [23,24]. Moreover, Nicolas conducted an in vitro investigation involving the sliding of both shod and unshod horse hooves across various surfaces, such as concrete, pavement, asphalt, and rubber. The meticulous measurement of traction forces was carried out using a portable digital force meter and a force plate. This study revealed that the coefficient of friction for horse hooves consistently fell within the range of 0.5–0.8 [25].

### 2.5. Simulation Results with Ungula and Frozen Soil

Figure 2 shows the stress distribution between ungula and frozen soils under conditions of stable friction. The stress of frozen soil is concentrated at 0.53–3.17 MPa (Figure 2D), and the stress of ungula is concentrated at 4.56–24.72 MPa (Figure 2E). During trotting, the reindeer ungula received a large reaction force from the ground, and the ungula was embedded in the frozen soil, which improved the attachment performance of the ungula and played an antislip role.

## 3. Microstructures of Plantar Tissues

### 3.1. Sample Processing

The plantar fur of reindeer is located below the ungula, partially wrapping the ungula, and directly contacts the frozen soil, providing effective protection and attachment. The ungula cusp, outer edges, and fur tissues were extracted from the right front hooves of the reindeer. The samples were placed in a vacuum box and plated with gold (Edwards sputter coater S150 B) and detected with a 1000 B scanning electron microscope (SEM, Philips XL30 ESEM-FEG, Berlin, Germany) operated at 5.0 KV.

### 3.2. Microstructures

**Figure 3:**
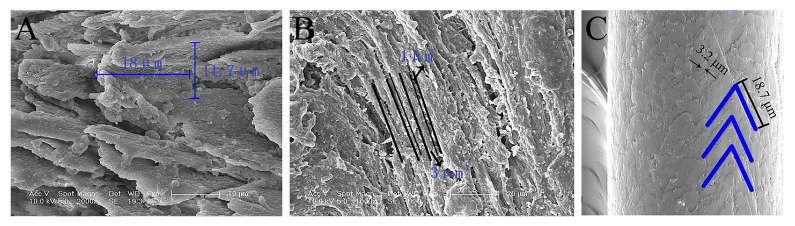
Microstructure of the stance unit in reindeer. (**A**) Ungula cusp; (**B**) ungula outer edge; (**C**) plantar fur.

For reindeer in winter, the stance unit of the feet is the only part in contact with frozen soil [26]. Microscopically, the ungula cusp of the reindeer had a serrated rib structure and was severely worn, with many cracks. The ribs on the outer edges were distributed longitudinally with a rough surface. According to the specific size of the ungula cusp ribs, the rib width and length were measured, and the corresponding ratio was 0.65:1 (Figure 3A). According to the size of the ungula outer edge ribs, the rib width and distance were measured, and the corresponding ratio was 3:1 (Figure 3B). In addition, the scales of the plantar fur were very tightly arranged and had large ripples, and the ratio of width to length of the scales was approximately 0.17:1 (Figure 3C). These special structures may play a role in the attachment of reindeer moving on frozen soil.

## 4. Bionic Foot Design, Processing, Strength Check, and Attachment Test

### 4.1. Bionic Design

#### 4.1.1. Anterior of a Bionic Ribbed Foot

**Figure 4:**
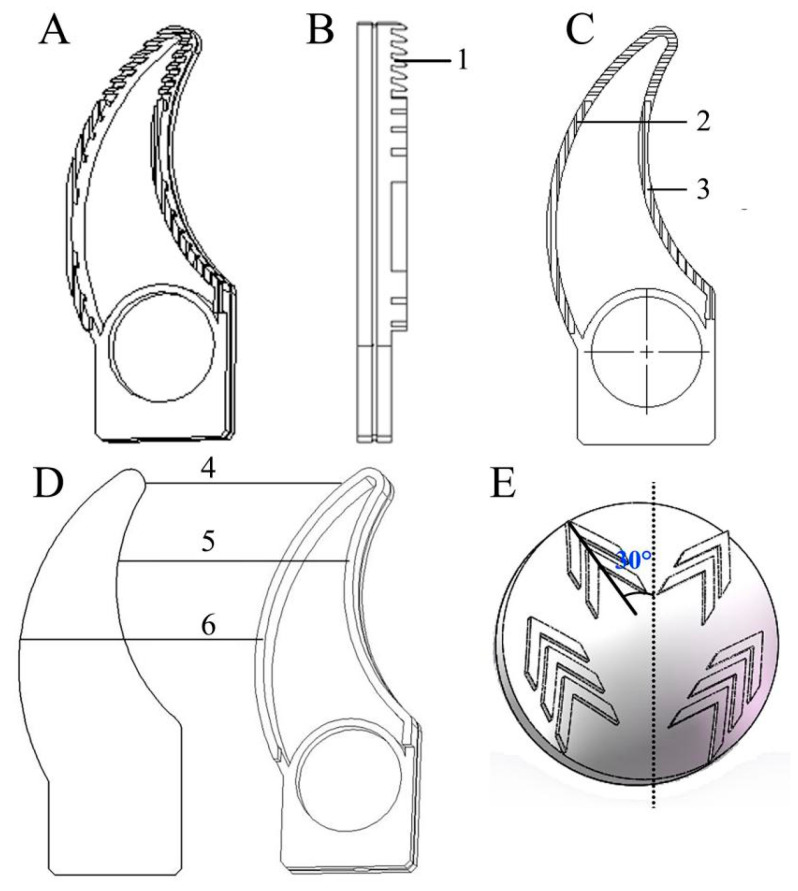
Design of the bionic ribbed foot anterior, bionic ribless foot anterior, and ungula capsule. (**A**) Medial view of the bionic ribbed foot anterior, (**B**) lateral view of the bionic ribbed foot anterior; and (**C**) plantar view of the bionic ribbed foot anterior; (**D**) three-dimensional model of the bionic ribless foot anterior; (**E**) three-dimensional structure of the bionic ungula capsule. 1—ungula cusp (serrated rib), 2—ungula outer edge (longitudinal rib), 3—ungula inner edge (longitudinal rib), 4—curve of ungula cusp, 5—curve of ungula inner edge, 6—curve of ungula outer edge.

The bionic design concept and the bionic ribbed parts at the ungula cusp and ungula outer edge were designed. The anterior of the bionic ribbed foot consists of the serrated ribs at the cusp and the longitudinal ribs at the outer edge (Figure 4A–C).

#### 4.1.2. Anterior of the Bionic Ribless Foot

The cusp, outer edge, and capsule of the ungula play the main attachment roles during the movement of reindeer. The ungula cusp curve was extracted from the reconstructed model of reindeer ungula to provide the design basis for the bionic attachment foot. The ungula cusp curve equation:

$$y=-1.81-2.22x+0.25x^{2}-0.01x^{3}\;(R^{2}=93.03\%)$$

Given the smoothness of the foot structure, the inner and outer edge curves were extracted. The inner edge curve equation:

$$y=1.04+0.97x-0.01x^{2}+1.92\times 10^{-5}x^{3}\;(R^{2}=99.74\%)$$

The outer edge curve equation:

$$y=0.75+0.42x-2.79\times 10^{-3}x^{2}-1.86x^{3}\;(R^{2}=99.74\%)$$

After the ungula cusp and inner and outer edge curves were moved inward by an equidistant of 3.6 mm, the curves were stretched by 18.0 mm, and the internal groove was stretched by 1.2 mm to establish a three-dimensional model of the bionic ribless structure (Figure 4D).

#### 4.1.3. Design of a Bionic Ungula Capsule

The reconstructed model of the ungula capsule was converted into a three-dimensional model. In order to ensure the suitable density and reasonable distribution of the bionic pattern, the sizes of the ungula capsule and plantar fur patterns were collected. We arranged four and six bionic patterns on the distal and proximal ends of the bionic ungula capsule, respectively. Given the angle of the plantar fur pattern, the bionic pattern was distributed to both sides at an angle of ~30° to the center line of the ungula capsule (Figure 4E).

#### 4.1.4. Comparative Design of Multidamboard Foot

**Figure 5:**
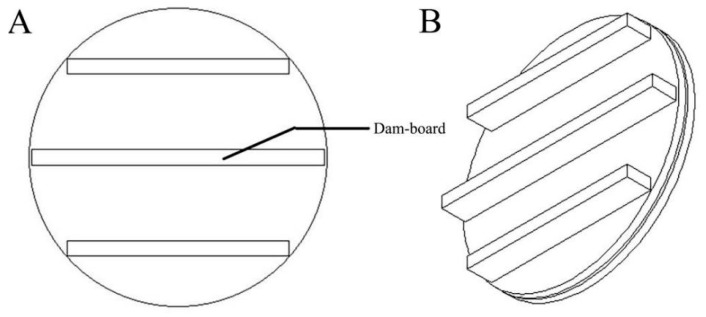
Three-dimensional structure of the multidamboard foot. (**A**) Plantar view; (**B**) medial view.

Reportedly, the attachment ability of the multidamboard foot was good [27]. Combined with the specific dimensions of the bionic foot, a multidamboard foot was designed, and its plantar view and lateral view are shown in Figure 5. The bionic foot is consistent with the ordinary multidamboard foot in height, upper surface area, and lower surface contact area (Table 1).

### 4.2. Strength Check of Bionic Foot

**Figure 6:**
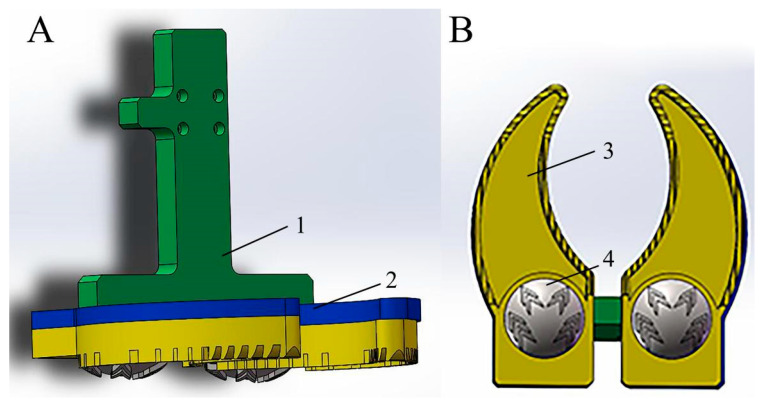
Bionic foot model. (**A**) Medial view; (**B**) plantar view. 1—slider connecting plate, 2—foot connecting block, 3—load-bearing toe, and 4—bionic ungula capsule.

The bionic foot with ribs and an ungula capsule (bionic foot 2) was finally applied to the mechanical foot. The slider connecting plate can connect the stance units (foot connecting block, bionic ungula capsule, and load-bearing toe) and the upper mechanical foot (Figure 6A,B). To analyze the strength of the slider connecting plate of the bionic mechanical foot, an impact simulation was established between the simplified foot and frozen soil.

#### 4.2.1. Simulation Parameters and Conditions

With the Abaqus/Explicit dynamic display solving method, when a bionic foot was in impact contact with frozen soil, the slider connecting plate and the foot connecting block were set to stainless steel, and the bionic ungula capsule and load-bearing toe were set to hard rubber and ABS, respectively. Then, the stance units (slider connecting plate, foot connecting block, bionic ungula capsule, and the load-bearing toe) were ribbed. The tangential behavior of the interaction between the frozen soil and the bionic foot was set as the friction coefficient, and the frozen soil was fixed. The mechanical foot carries out horizontal traction and can move up and down in a vertical direction when in contact with the frozen soil. According to previous mechanical experiments on the foot, the peak force experienced by the bionic foot was 200–400 N. Therefore, the impact contact time between the mechanical foot and the ground was set to be 0.01 s, and the impact load was set to be 300 N. The specific material parameters are shown in Table 2.

#### 4.2.2. Strength Analysis

**Figure 7:**
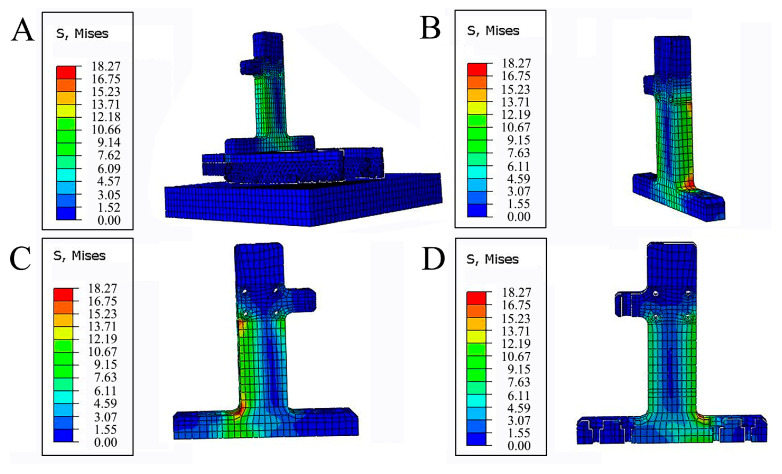
Stress distribution at the mechanical foot. (**A**) Stress distribution of each structure; (**B**) stress distribution in the medial view of the connecting plate; (**C**) stress distribution in the lateral view of the connecting plate; (**D**) stress distribution in the cross-sectional view of the connecting plate.

The stress distributions of each structure at the mechanical foot are shown in Figure 7. The stress distributions at the foot connecting block, the bionic ungula capsule, and the load-bearing toe were relatively uniform, and there was an obvious stress concentration on the slider connecting plate. Specifically, the stress of frozen soil is concentrated at 0.41–1.22 MPa, the stresses of the foot connecting block, the bionic ungula capsule, and the load-bearing toe are concentrated at 1.72–5.05, 5.81–17.39, and 1.70–5.09 MPa, respectively, while the stress of the slider connecting block is larger and concentrated at 6.11–18.27 MPa. The yield strength and tensile strength of the steel were approximately 235 and 645 MPa, respectively [31,32]. During the impact process, the maximum stress at the corner of the slider connecting plate was approximately 28 MPa. Although the slider connecting plate generated a stress concentration at the corner, its maximum stress was much lower than the yield strength and tensile strength of the steel. Therefore, the strength of the mechanical foot complies with the test requirements.

### 4.3. Attachment Experiment on Frozen Soil

#### 4.3.1. Engineering Processing

The anterior of the bionic foot and the bionic ungula capsule were both processed by 3D printing using ABS-M30 and rubber, respectively. ABS-M30 is a strong and durable engineering plastic. The hardness of rubber printing materials is mostly at Shore A40–95.

**Figure 8:**
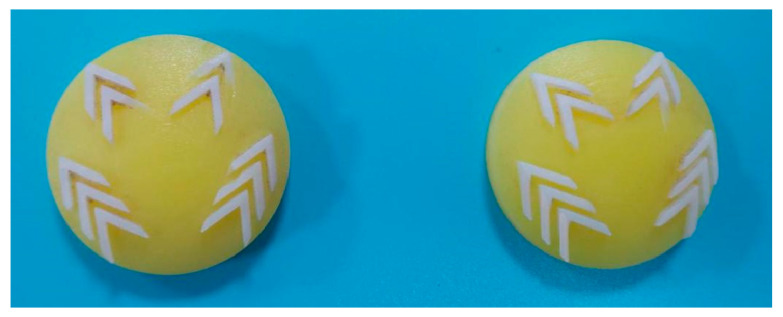
Bionic ungula capsule.

Given the different hardness required for bionic ungula capsules and bionic patterns, as well as the hardness of the rubbers available on the market, an LX-A Shore hardness tester (range: 0–100 HA, accuracy: <1% H) was used to test the rubber materials, as shown in Table 3. Shore A60 rubber was used for the bionic ungula capsule, and Shore A85 rubber was used for the bionic patterns (Figure 8).

**Figure 9:**
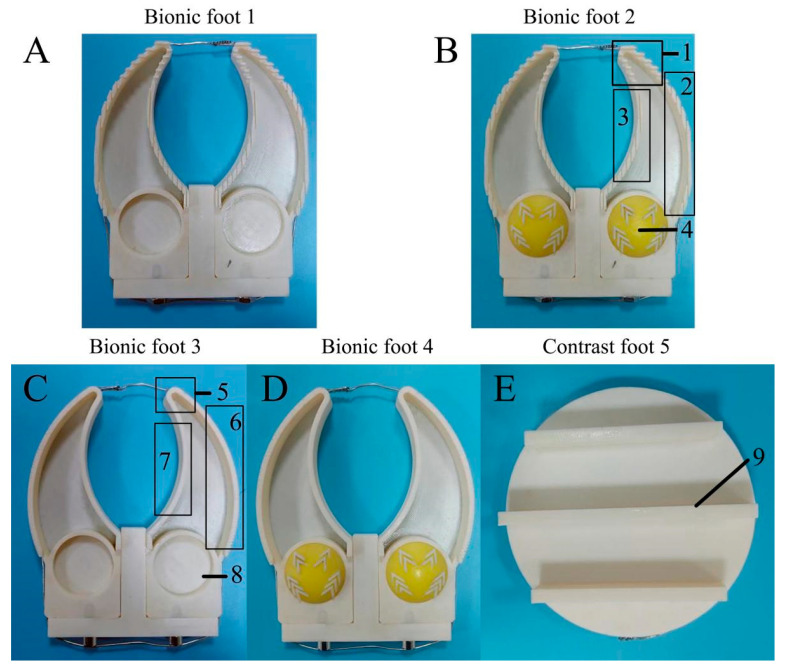
Attachment feet. (**A**) Bionic foot with ribs and no ungula capsule; (**B**) bionic foot with ribs and ungula capsule; (**C**) bionic foot without ribs and ungula capsule; (**D**) bionic foot without ribs and with ungula capsule; (**E**) multidamboard foot. 1—ungula cusp (serrated rib), 2—ungula outer edge (longitudinal rib), 3—ungula inner edge (longitudinal rib), 4—bionic ungula capsule, 5—without cusp ribs and ungula capsule, 6—without outer edge ribs, 7—without outer inner ribs, 8—without ungula capsule, 9—dam-board.

The bionic foot with ribs and no ungula capsule is composed of a bionic ribbed foot (bionic foot 1), and the bionic foot with ribs and ungula capsule is composed of a bionic ribbed foot and a bionic ungula capsule (bionic foot 2). The bionic ribless foot, without an ungula capsule, is composed of a bionic ribless foot (bionic foot 3). The bionic foot without ribs and with an ungula capsule is composed of a bionic ribless foot and a bionic ungula capsule (bionic foot 4). Moreover, a conventional multidamboard foot of the same size was designed (contrast foot 5) (Figure 9).

#### 4.3.2. Friction Testing Machine

**Figure 10:**
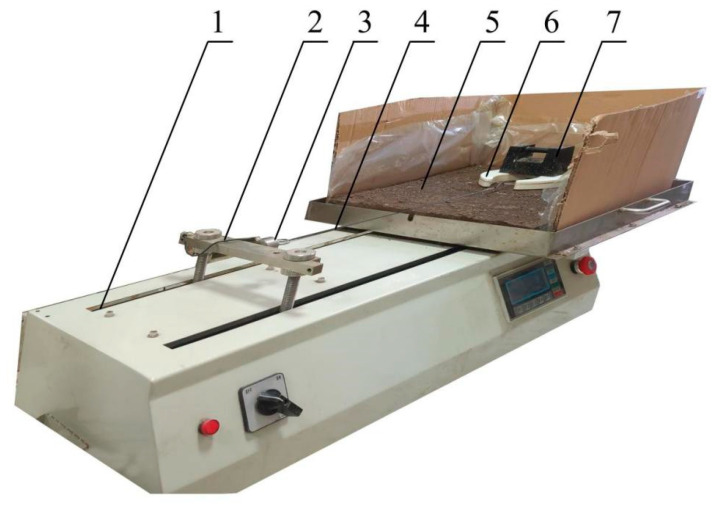
Frozen ground attachment test. 1—track, 2—lifting rod, 3—tension sensor, 4—traction line, 5—frozen ground, 6—bionic foot, 7—weight.

Frozen ground attachment experiments were performed with a UTM friction testing machine (Figure 10). The tray with frozen ground was fixed to the testing machine. One end of the traction line was connected to the attachment foot, and the other end was connected to the tension sensor on the lifting rod. The sensor’s accuracy was 0.0001. The position of the lifting rod was adjusted to keep the traction line horizontal. After the lifting rod was fixed, it moved horizontally and uniformly on the electric track. The moving speed range and maximum moving distance were 10–500 mm/min and 200 mm, respectively.

#### 4.3.3. Experimental Conditions

The experimentation process was conducted in a cold room with the temperature controlled at approximately −10 °C. During the experiment, the room was closed to avoid the influence of the wind, and the surface temperature of the frozen ground was stable at ±0.5 °C. The signals of friction, force, and displacement were detected using force and displacement sensors and transmitted to a computer. As the only part in contact with the frozen ground, the attachment foot needs to provide all the reaction force for the movement of the mechanical foot, which is similar to the attachment force on the surface of a vehicle wheel. Therefore, Coulomb’s law was introduced to calculate the dynamic coefficient of friction (DCOF), which was defined as the ratio of the dynamic friction force *F* to the normal load *N*.

For each experimental condition, we conducted five repeated experiments for each type of foot. The DCOFs of the five feet were studied under the same moving speed and normal load. Due to the limitations of the test conditions, the normal load cannot be too large, so we set the pressure to be 20 N. A preliminary friction test was conducted at a speed of 50–150 mm/min to determine the horizontal movement speed. When the moving speed was lower than 80 mm/min, the acquisition curve fluctuated greatly. When the moving speed exceeded 120 mm/min, the data sampling frequency was too low, resulting in partial data loss. When the moving speed was 100 mm/min, the acquisition curve was stable, and the sampling frequency was moderate. Thus, 100 mm/min was selected as the test speed. After each test, the frozen ground was wiped with sandpaper to keep the surface smooth and clean. After every five tests, the ground was replaced to prevent cracks on the frozen ground from affecting the test.

In our experiment, we applied a fixed pressure of 20 N across all tests to ensure consistency and facilitate direct comparisons between different bionic feet. However, it is important to note that this fixed pressure value simplifies the dynamic and variable pressures experienced by a reindeer foot during locomotion across diverse terrains. Real-world pressures on a reindeer foot can vary significantly, especially during activities like trotting, depending on factors such as body weight, movement speed, and substrate nature. Our fixed pressure condition does not account for these variations. Researchers should exercise caution when extrapolating our results to real-world reindeer locomotion.

#### 4.3.4. Undisturbed Frozen Soil Attachment Test

**Figure 11:**
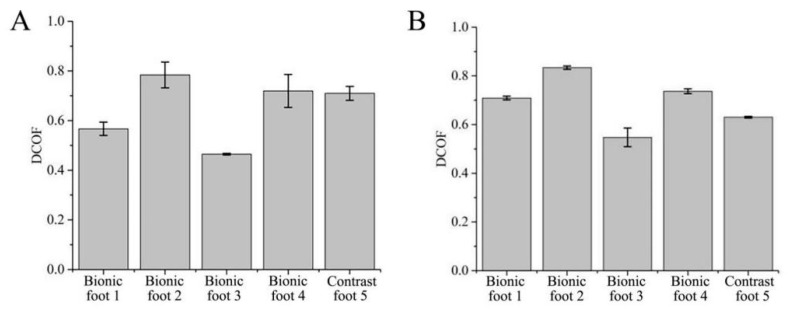
Comparison of the attachment properties of the five feet on undisturbed frozen soil. (**A**) Loose state (soil frozen in loose state); (**B**) compact state (soil frozen in compact state).

The values and variation patterns of the DCOFs of the five feet on frozen soil (16.4% moisture) are shown in Figure 11. The DCOFs of the five feet on soft frozen soil rank as bionic foot 2 > bionic foot 4 > contrast foot 5 > bionic foot 1 > bionic foot 3 (Figure 11A). The DCOFs of the five feet on tight original frozen soil rank as bionic foot 2 > bionic foot 4 > bionic foot 1 > contrast foot 5 > bionic foot 3 (Figure 11B). The bionic foot (foot 2 and foot 4) played a good attachment role. Compared with the multidamboard foot 5, the ribbed and ungula capsule bionic foot 2 and the ribless ungula capsule bionic foot 4 can increase DCOF by 11.43–31.75% and 2.86–17.46%, respectively.

**Figure 12:**
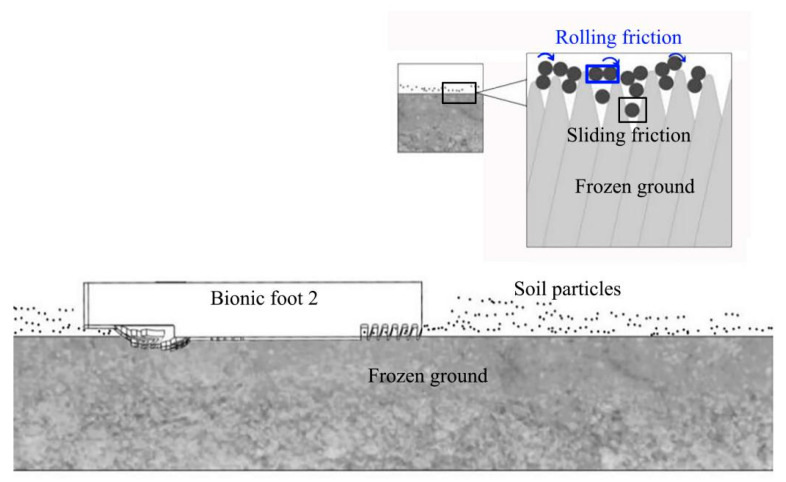
The mechanism of interaction between the bionic foot and the undisturbed frozen soil.

Based on the observed results and the analyses of the experimental data, when interacting with the undisturbed frozen soil, the bionic foot with ribs and ungula capsule (bionic foot 2) can be embedded into the frozen soil (Figure 12). The bionic foot 2 increased the embedded depth through the bionic ungula capsule, and the anterior of the bionic ribbed foot had better traction performance. There were many soil particles unconsolidated with ice distributed on the surface of the undisturbed frozen soil. When the foot interacts with the frozen soil, they can be embedded into the soil, further increasing the soil particles on the surface of the frozen soil. This part of the soil particles began to roll upon the interaction with the foot, and the frozen soil generated rolling friction with the foot, which reduced the attachment performance of the foot.

## 5. Conclusions

Based on the motion speed and ground pressure data of reindeer hooves during trotting, the friction between the ungula and the frozen ground during trotting was simulated. Based on scanning electron microscopy (SEM), the macro/microstructures of plantar fur were characterized, and the attachment mechanism between the sole unit and the frozen ground was studied. When the reindeer interacted with the frozen ground while trotting, the stress distribution on the ground was concentrated on the ungula cusp, ungula outer edge, and ungula capsule. These parts play a role in attachment. When the reindeer moved, both the proximal and distal ends of the ungula were in contact with the ground at the early stance stage. At this time, the parts of the hooves that played a role in load bearing were mainly the ungula cusp, ungula outer edge, and ungula capsule. In the latter stance stage, only the distal end of the ungula was in contact with the ground. At this time, the hooves were pushing on the ground, and the parts responsible for the attachment function were mainly the ungula cusp and ungula outer edge.

Under the macro-micro synergy of the plantar fur and ungula morphology, the stance unit of reindeer plays an attachment role. With the reindeer hoof stance unit as the bionic prototype and based on the typical curves of ungula, ungula capsule curved surface, microstructures, and the nonsmooth surface of plantar fur, four bionic attachment feet and an ordinary multidamboard foot were designed. When interacting with soft or tight frozen soil, the ribbed and ungula capsule bionic foot 2 showed the best attachment performance, followed by the ribless and ungula capsule bionic foot 4. Compared with the multidamboard foot 5, bionic foot 2 and foot 4 increased DCOF by 11.43–31.75% and 2.86–17.46%, respectively, under all frozen soil conditions. The attachment mechanism is that, under the action of pressure, the small patterns of the bionic ungula capsule rub against the nonsmooth structure of the frozen soil surface, which improves the attachment performance. At the same time, the bionic ribbed anterior of the foot can provide better traction, so bionic foot 2 has the best attachment performance.

Based on the features of reindeer hooves, along with the macro/microstructures and attachment mechanism of the stance unit, the investigation into the design, traction, and attachment performance of bionic feet has provided valuable insights. This study not only contributes to our understanding of attachment mechanisms between bionic feet and challenging terrains like frozen ground, but also lays the groundwork for the development of advanced bionic feet. In addition, our study sets the stage for the development of bionic robotic systems, particularly on frozen ground. The macro/micro synergy observed between plantar fur and ungula morphology presents a unique opportunity for designing bionic feet that exhibit enhanced attachment performance. This has potential applications in fields such as search and rescue operations, planetary exploration, and other scenarios where robots need to traverse challenging frozen ground.

## Figures and Tables

**Table 1:** Specific size of the bionic foot and multidamboard foot.

	Height (mm)	Upper Surface Area (mm^2^)	Lower Surface Contact Area (mm^2^)
Bionic foot	25.00	14,307.10	2324.00
Multidamboard foot	25.00	14,224.20	2326.21

**Table 2:** Parameters of model geometry and materials.

Model	Density ρ (t/mm^3^)	Elastic Modulus E (MPa)	Poisson’s Ratio γ	Data Source
Stainless steel	7.85 × 10^−9^	2.06 × 10^5^	0.30	[28]
ABS	1.10 × 10^−9^	2.20 × 10^3^	0.40	[29]
Hard rubber	3.00 × 10^−9^	13.23	0.47	Material tests
Frozen soil	1.80 × 10^−9^	34.12	0.29	Material tests and [30]

**Table 3:** Measured results of rubber hardness.

Hardness											Mean ± Deviation
Soft rubber	60	62	61	59	58	60	56	64	62	58	60 ± 2.4
Hard rubber	88	85	85	82	85	87	83	84	89	82	85 ± 2.4

## Data Availability

Data will be made available upon request.
